# AI revolution in insurance: bridging research and reality

**DOI:** 10.3389/frai.2025.1568266

**Published:** 2025-04-09

**Authors:** Sukriti Bhattacharya, German Castignani, Leandro Masello, Barry Sheehan

**Affiliations:** ^1^Human-Centered AI, Data and Software (HANDS), Luxembough Institute of Science and Technology, Maison de l'innovation, Esch-sur-Alzette, Luxembourg; ^2^Kemmy Business School, University of Limerick, Limerick, Ireland

**Keywords:** artificial intelligence, regulations, health insurance, property insurance, automotive insurance, PRISMA

## Abstract

This paper comprehensively reviews artificial intelligence (AI) applications in the insurance industry. We focus on the automotive, health, and property insurance domains. To conduct this study, we followed the PRISMA guidelines for systematic reviews. This rigorous methodology allowed us to examine recent academic research and industry practices thoroughly. This study also identifies several key challenges that must be addressed to mitigate operational and underwriting risks, including data quality issues that could lead to biased risk assessments, regulatory compliance requirements for risk governance, ethical considerations in automated decision-making, and the need for explainable AI systems to ensure transparent risk evaluation and pricing models. This review highlights important research gaps by comparing academic studies with real-world industry implementations. It also explores emerging areas where AI can improve efficiency and drive innovation in the insurance sector. The insights gained from this work provide valuable guidance for researchers, policymakers, and insurance industry practitioners.

## 1 Introduction

The insurance industry has a long history of adapting to technological advancements, from standardized actuarial risk models to the introduction of digital automation (Pearson, 2015). Today, Artificial Intelligence (AI) drives this transformation, enabling efficiency gains in underwriting, claims processing, and fraud detection while addressing sustainability challenges and regulatory complexity (McKinsey, 2022; Eling et al., 2022). Insurers now face dual pressures: achieving operational efficiency through AI and meeting stringent ethical and regulatory requirements and emerging governance frameworks (Eurofi Magazine, 2024; Habib, 2024). These demands necessitate AI systems that balance predictive power with transparency, particularly as climate-related risks and data privacy concerns reshape the industrys priorities (Li and Guo, 2024; Mahadik et al., 2025).

Despite numerous studies on AI applications in specific insurance sectors–such as automotive, health, and property insurance (Adeoye et al., 2024; Srivastava et al., 2024; Eckert and Osterrieder, 2020; Kumar et al., 2019; Maier et al., 2020; Balasubramanian et al., 2018; Ali et al., 2022; Mashrur et al., 2020; Krefting et al., 2023)–existing research lacks a comprehensive, systematic review that evaluates AI's integration across the entire insurance industry. Previous studies have typically focused on single-domain implementations or fragmented applications, without addressing cross-sectoral adoption trends, challenges, or regulatory constraints. This gap limits stakeholders' ability to benchmark AI's holistic impact on risk governance and sustainability (Habib and Mourad, 2024). A PRISMA-guided systematic review is critical to consolidate insights, address methodological inconsistencies, and identify interoperable solutions for multi-domain integration (Sarkis-Onofre et al., 2021; Page et al., 2021).

The urgency of this research stems from three industry-specific challenges. First, insurers increasingly rely on opaque AI models for risk assessment and pricing, raising ethical concerns about bias and exclusion (Charpentier, 2024; Oguntibeju, 2024). Second, regulatory frameworks demand explainable AI to ensure compliance and public trust, yet most implementations remain black-box systems (Ramesh, 2025). Third, sustainability goals require AI architectures that optimize resource allocation while adapting to dynamic risks like climate change (Mahadik et al., 2025). Current literature inadequately addresses these intersecting priorities, leaving a critical disconnect between academic prototypes and scalable, ethically aligned industry solutions.

This study aims to bridge the gap between academic research and industry practices by conducting a systematic PRISMA-based review of AI applications across automotive, health, and property insurance. The key objectives of this research are:

1. To provide a comprehensive analysis of AI applications across different insurance domains, focusing on risk assessment, fraud detection, claims processing, and policy personalization.
2. To identify the key insurers and InsurTech companies actively adopting AI solutions, evaluating their AI-driven services and innovations.
3. To examine critical challenges in AI adoption, including regulatory compliance, ethical concerns, explainability, and data governance.
4. To highlight future research directions by identifying gaps in existing AI methodologies and their impact on insurance sustainability.

By synthesizing academic and industry perspectives, this review provides actionable insights for policymakers shaping AI regulations, insurers balancing efficiency with ethical AI, and researchers designing adaptable systems for emerging risks.

This paper is structured to provide a comprehensive and systematic review of the application of artificial intelligence (AI) in the insurance industry, with a focus on automotive, health, and property insurance domains. Section 2 investigates the theoretical background, exploring the foundational frameworks that underpin the integration of AI in insurance, including technology acceptance and adoption, risk management and decision-making, sustainable and responsible AI, and cognitive and behavioral dimensions. Section 3 presents a detailed literature review of AI applications in insurance, categorized by academic research and industry-wide adoption, highlighting key findings and methodologies across different insurance sectors. Section 4 outlines the methodology employed for this systematic review, following the PRISMA guidelines to ensure rigorous and unbiased analysis. Section 5 discusses the significant findings from the review, comparing academic research with industry implementations and identifying key gaps and opportunities. Section 6 provides a critical discussion of the theoretical and managerial implications of the findings, outlines limitations, and suggests a future research agenda. Finally, Section 7 wraps everything up with a conclusion. We summarize the key points and reiterate why this research is important for anyone interested in how AI is changing the insurance industry.

## 2 Theoretical background

The integration of artificial intelligence (AI) in the insurance sector draws from several established theoretical frameworks that span technology adoption, risk management, and sustainable business practices. These interconnected foundations help explain how AI is reshaping traditional insurance operations. Our work builds on the following theoretical foundations by examining AI implementation across insurance value chains. We investigate how these technologies enhance efficiency and decision quality while addressing emergent challenges in regulatory compliance, ethics, and organizational adaptation. By connecting theoretical frameworks with empirical evidence, we contribute to a more textured understanding of AI's role in transforming insurance markets and practice.

### 2.1 Technology acceptance and adoption

The willingness of insurance organizations to implement AI solutions is deeply influenced by the perceptual factors described in Davis's technology acceptance model Davis (1989). When insurance executives evaluate AI tools for underwriting or claims processing, their perceptions of usefulness and ease of implementation often determine whether these technologies gain traction within their organizations. Recent research by Gabelaia et al. (2024) has extended this model to account for the unique challenges of deploying complex AI systems in risk-sensitive environments.

The adoption of AI across the insurance landscape follows patterns consistent with Rogers's Diffusion of Innovation Theory (Rogers et al., 2014), where technology-oriented firms pioneer implementation before practices spread throughout the industry. This phenomenon is particularly evident in the uneven adoption of machine learning algorithms across different market segments and regions (Eling and Lehmann, 2018). As Puschmann (2017) notes, this diffusion is accelerated when early adopters demonstrate measurable returns on their AI investments.

### 2.2 Risk management and Decision-Making

Insurance risk management's theoretical foundations create natural alignment with AI capabilities. The Expected Utility Theory (Von Neumann and Morgenstern, 2007) explains how insurers traditionally approach probabilistic risk evaluation. AI systems enhance these approaches by identifying subtle patterns in enormous datasets that would elude human analysts.

Kahneman and Tversky's Prospect Theory (Kahneman and Tversky, 2013) illuminates the human dimensions of risk assessment and decision-making in insurance. Their work helps explain why policyholders often struggle with AI-generated risk assessments, particularly when these contradict intuitive judgments or established practices. Building on this foundation, Gigerenzer and Brighton (Gigerenzer and Brighton, 2009) argue that successful AI integration must acknowledge the heuristic nature of human decision-making rather than imposing purely algorithmic rationality on insurance processes.

### 2.3 Sustainable and responsible AI in insurance

Carroll's Corporate Social Responsibility framework (Carroll, 1991) offers a valuable lens for examining ethical AI implementation in insurance. Keller (2020) argues that responsible AI deployment must balance profit motives with fairness, accountability, and transparency. It reveals significant reputation benefits for insurers who proactively address algorithmic bias.

Regulatory frameworks increasingly shape how insurers develop AI systems. While the European AI Act (European Commission, 2023) establishes clear guidelines, Wilson and Daugherty (2018) argue that successful regulation must focus on human-AI collaboration rather than treating these systems as autonomous decision-makers, a principle increasingly reflected in insurance governance frameworks.

### 2.4 Cognitive and behavioral dimensions

The behavioral economics research pioneered by Thaler and Sunstein? provides crucial insights into how AI-mediated insurance services influence consumer choices. Bhargava and Loewenstein (2015) extend this analysis specifically to insurance decision-making, revealing how choice architecture in digital environments shapes coverage selection and risk perception. Their work suggests that AI interfaces can potentially reduce known behavioral biases in insurance purchasing.

The psychological dimensions of human-AI interaction find further grounding in Nass and Moon (2000)'s research on social responses to technology. Expanding on this foundation, Lee and See (2004) explore how trust calibration affects human interaction with automated systems, while Castelo et al. (2019) examine how algorithm aversion manifests specifically in high-stakes financial decisions like insurance selection. Their findings suggest that perceived algorithmic transparency significantly impacts consumer comfort with AI-driven insurance processes.

## 3 Literature review and hypotheses development

### 3.1 Application of AI in insurance sectors-academic research

The following reviewed literature highlights AI's versatility and transformative potential in the automotive, property, and health insurance industries summarized in Table 1. The following sections contain the contributions of each paper, in brief, categorized into three different insurance sectors, automotive, health, and Property.

**Table 1:** AI applications in insurance (scientific papers).

**Ref**.	**References**	**Key Findings**	**Methodology**	**Coverage**	**Stakeholders**
1	Ayuso Mercedes (2019)	Insurance pricing and risk assessment	Telematics data integration	Auto	Underwriters
2	Gao et al. (2022)	Insurance pricing and risk assessment	Neural networks, telematics data integration	auto	Underwriters
3	Reimers and Shiller (2018)	Usage-based insurance analysis	AI on Telematics data	Auto	Insurers, Consumers
4	Huang and Meng (2019)	Usage-based insurance rate-making	Data binning, predictive models	Auto	Underwriters
5	Mullins et al. (2021)	Ethical considerations of AI in insurance	Transparency, explainability, governance	Auto, Health, property	Insurers, regulators
6	Rababaah (2023)	Vehicle damage detection	Convolutional Neural Networks (CNNs)	Auto	Insurers, clients
7	Meenakshi and Sivasubramanian (2023)	Vehicle damage detection	Deep neural networks	Auto	Insurers, clients
8	Fouad et al. (2023)	Vehicle damage detection	Pre-trained CNNs (VGG-19, DenseNet-169)	Auto	Insurers, clients
9	Samarasinghe et al. (2021)	Vehicle damage detection	Convolutional Neural Networks (CNNs)	Auto	Insurers, clients
10	Sree et al. (2023)	Vehicle damage detection	Convolutional Neural Networks (CNNs)	Auto	Insurers, clients
11	Sinha et al. (2021)	Agentless insurance solutions	Statistical machine learning models	Auto	Insurers
12	Nanda et al. (2023)	End-to-end insurance automation	AI, Blockchain, Instance segmentation	Auto	Insurers, clients
13	Benedek and Nagy (2023)	Fraud detection comparison	AI-based vs. traditional methods	Auto	Insurers
14	Mahohoho et al. (2023)	Automated loss reserving	Machine learning algorithms	Auto, Health, Property	Actuaries
15	Samiuddin et al. (2023)	Health insurance risk assessment	Deep Neural Network (DNN)	Health	Underwriters, Insurers
16	Kaushik et al. (2022)	Health insurance premium prediction	Artificial Neural Network (ANN)	Health	Underwriters, clients
17	Orji and Ukwandu (2024)	Health insurance cost prediction	XGBoost, GBM, Random Forest	Health	Underwriters, clients, policymakers
18	Kapadiya et al. (2022)	Health insurance fraud detection	Machine learning models	Health	Insurers, clients
19	Thesmar et al. (2019)	Health insurance data analysis	Machine learning, claims data integration	Health	Insurers, policymakers
20	Isa et al. (2022)	Health insurance data analysis	AI for health financing	Health	Insurers, policymakers
21	Ramezani et al. (2023)	Health insurance data analysis	AI for health financing	Health	Insurers, policymakers
22	Sessional Meeting Discussion Balboa et al. (2022)	Health insurance risk assessment	AI for mental health risk assessment	Health	Underwriters, clients
23	Bora et al. (2022)	Health insurance premium explanation	Explainable AI (LIME, SHAP)	Health	Clients, underwriters
24	Rajagopal (2020)	Property insurance risk assessment	AI, predictive analytics	Property	Underwriters
25	Chen et al. (2022)	Property insurance risk assessment	AI for blueprint analysis	Property	Underwriters
26	Dabbugudi (2022)	Property insurance pricing	Neural networks, NLP	Property	Underwriters
27	Severino and Peng (2021)	Property insurance fraud detection	Ensemble classifiers, deep neural networks	Property	Fraud investigators
28	Shi et al. (2016)	Property insurance fraud detection	Copula regression	Property	Fraud investigators
29	Barr et al. (2017)	Home valuation	Gradient boosting	Property	Underwriters
30	Quijano Xacur and Garrido (2015)	Property insurance pricing	Generalized linear models	Property	Underwriters
31	Radu and Alexandru (2022)	Process optimization, customer experience	Machine learning-powered virtual assistants	Property	Clients, agents
32	D'Arcy and Gorvett (2004a)	Strategic decision-making	Dynamic financial analysis, AI techniques	Property	Underwriters, managers
33	Guillen et al. (2021)	Penalization of near-miss events to promote safe driving behavior	AI models and statistics	Auto	Underwriters, policymakers
34	Li et al. (2023)	Long-term risk reduction	AI and statistical analysis	Auto	Underwriters, policymakers
35	Ma et al. (2018)	Integrating real-time GPS data into automotive insurance pricing	AI and statistical analysis	Auto	Underwriters, policymakers
36	Masello et al. (2023)	Improve risk assessment in automotive insurance by considering factors like weather, traffic, and road conditions	Explainable AI	Auto	Underwriters, policymakers

#### 3.1.1 Automotive insurance applications

The application of AI in the automotive insurance industry has been a topic of growing interest and innovation. Researchers have explored various ways to leverage these technologies to enhance insurance pricing, risk assessment, claims processing, and operational efficiency. In this context, we review the key contributions of recent research papers based on the following categorizations.

##### 3.1.1.1 Insurance pricing and risk assessment:

The paper by Ayuso Mercedes (2019) highlights the importance of the factors related to driver behavior and risk exposure in insurance pricing models. By incorporating telematics data, such as ‘mileage driven', ‘speed limits exceeded', and ‘types of roads frequented', insurers can better understand individual driver risk profiles. This allows for more personalized and accurate premium calculations that improve pricing accuracy and risk assessment. Similarly, the paper by Gao et al. (2022) enhances classical actuarial regression models for claim frequency prediction by incorporating telematics car driving data. The authors utilize data-driven neural network approaches, including a densely connected feed-forward neural network and a convolutional neural network, to process the telematics data. The authors integrate it with traditional risk factors, demonstrating the synergistic effect for achieving the best predictive models in automotive insurance.

##### 3.1.1.2 Telematics and Usage-based Insurance (UBI):

The paper by Reimers and Shiller (2018) provides an empirical analysis of the impact of “Pay How You Drive” (PHYD) insurance programs on fatal accidents and profits in the automotive insurance industry. The study offers insights into the effects of proprietary data collection through telematics devices on market competition, consumer behavior, and road safety. Additionally, the paper by Huang and Meng (2019) focuses on the development of a comprehensive rate-making framework for Usage-based Insurance (UBI) products. The framework effectively combines data binning techniques (discrete binning) with various predictive models to enhance risk classification and predict claim frequency. The paper by Sinha et al. (2021) proposes a Software Application utilizing four Statistical Machine Learning Models to address specific tasks in the automotive insurance sector, such as predicting customer behavior, detecting fraud, and recommending policies, to replace human agents with AI algorithms. Similarly, Nanda et al. (2023) aim to streamline and automate the end-to-end insurance process, from user registration to claim settlement, by leveraging AI and blockchain technology, including an instance segmentation machine learning model for automated damage assessment. Guillen et al. (2021) introduce a new way to use telematics data for automotive insurance. They integrate telematics data into insurance pricing schemes, emphasizing the penalization of near-miss events to promote safe driving behavior. They combine the baseline insurance premium with additional charges for risky driving behaviors, such as “hard braking”, “acceleration events”, and “smartphone use” while driving, which ultimately impact the cost of insurance. Ma et al. (2018) explore integrating real-time GPS data into automotive insurance pricing. They use traffic app data and surveys to analyze driving behaviors and accident risks. Their key finding is that factors beyond traditional UBI metrics, like “speed compared to traffic flow”, are linked to accidents. In a recently published paper Li et al. (2023), authors propose a new approach for UBI programs that focuses on long-term risk reduction. It goes beyond simply reporting past behavior, instead uses advanced models to predict individual risk and offer personalized suggestions for improvement. The authors highlight the importance of individual-level analysis, arguing it allows for more targeted interventions and can be embedded in existing UBI frameworks. They validate their approach using real-world data and emphasize the actionable nature of the feedback provided to policyholders. Another recent paper by Masello et al. (2023) explores how telematics data, beyond driver behavior, can improve risk assessment in automotive insurance. The authors look at factors like weather, traffic, and road conditions to see how they influence risky driving events. They use machine learning to analyze a large dataset and identify the most important contextual factors. Their findings suggest that context significantly impacts risk and could be used to create a new insurance scheme based on driving location (‘Pay-where-you-drive'). They acknowledge privacy concerns with traditional telematics and propose context profiles as an alternative for insurers. This research also provides valuable insights for road safety efforts.

##### 3.1.1.3 Ethical considerations of AI in insurance:

The research paper by Mullins et al. (2021) provides a detailed analysis of the impact of AI, machine learning, and big data analytics on various levels within the insurance sector, including product-market dynamics, InsurTech operations, human-like AI capabilities, and the AI systems themselves. The paper emphasizes the importance of transparency, explainability, and governance in AI systems to address ethical issues effectively, highlighting the need to understand the intricate interactions between technical and social systems.

##### 3.1.1.4 Vehicle damage detection and assessment:

The paper by Rababaah (2023) presents a study of Deep Learning models of convolutional neural networks (CNN) applied to vehicle damage classification (VDC), achieving an impressive accuracy of 99.4% in classifying vehicle damages. Similarly, Meenakshi and Sivasubramanian (2023) explore different deep neural network-based techniques for vehicle damage detection, automating the damage assessment process with high accuracy confidence scores. Fouad et al. (2023) utilize adapted versions of pre-trained convolutional neural networks, specifically VGG-19 and DenseNet-169, for image classification to automate vehicle damage inspection, with DenseNet-169 outperforming VGG-19. Samarasinghe et al. (2021) apply Convolutional Neural Networks (CNNs) for image extraction in the implementation of an Enhanced Text Mining (ETM) system for Natural Language Processing, achieving 91% accuracy in identifying damaged vehicles. Sree et al. (2023) develop a Deep Learning approach, specifically Convolutional Neural Networks (CNNs), for vehicle damage detection and estimation, leading to efficient and automated damage assessment.

##### 3.1.1.5 Comparison of AI-based and traditional methods:

The paper by Benedek and Nagy (2023) challenges the prevailing notion that AI-based methods are inherently more cost-effective in fraud detection, providing valuable insights for decision-making in the insurance industry. The unexpected conclusion of their study was that the current AI-based automobile insurance fraud detection methods tested on real databases were found to be less cost-effective than traditional statistical-econometric methods. In contrast, the paper by Mahohoho et al. (2023) addresses the need for reliable estimates of claim costs and reserves in the general insurance sector, developing an Automated Actuarial Loss Reserving Model using eight machine learning algorithms, with Random Forests emerging as the best-performing model. The authors conclude that the model can improve the efficiency of claim settlement processes.

#### 3.1.2 Health insurance applications

AI is being applied in various aspects of the health insurance industry to improve risk assessment, personalize offerings, optimize operations, and enhance customer experiences. It's important to note that the application of AI in health insurance must be accompanied by robust data governance practices, ethical considerations, and adherence to privacy and security regulations, such as HIPAA in the United States, to ensure the protection of sensitive health information and maintain consumer trust. The following papers provide insights into the ways AI can be applied in health insurance to improve risk assessment, claims management, and overall efficiency.

##### 3.1.2.1 AI for health insurance risk assessment and premium prediction:

The paper by Samiuddin et al. (2023) addresses the challenge of accurately predicting health insurance costs based on individual characteristics, aiming to enhance the efficiency and precision of cost estimation in the healthcare industry. The study utilizes a Deep Neural Network (DNN) algorithm to predict medical insurance costs by training the model on attributes such as age, gender, body mass index, number of children, smoking habits, and location. The efficiency of the DNN model is demonstrated through its ability to quickly and accurately calculate costs compared to traditional machine learning algorithms. The main finding is that the DNN algorithm outperforms traditional machine learning algorithms in predicting health insurance costs. The conclusion highlights the potential of deep learning algorithms, particularly DNN, in revolutionizing cost prediction in the healthcare insurance sector, enabling insurance companies to develop more precise and timely insurance programs tailored to individual characteristics, leading to improved customer satisfaction and operational efficiency. The paper by Kaushik et al. (2022) addresses the prediction of health insurance premiums using a machine learning-based regression framework to accurately estimate health insurance costs for subscribers. The study utilizes an Artificial Neural Network (ANN)-based regression model to predict health insurance premiums, trained on a dataset containing relevant factors influencing insurance premiums, such as medical history, demographics, and lifestyle choices. The model achieves an overall accuracy of 92.72%. The main finding is the successful development and evaluation of an ANN-based regression model for predicting health insurance premiums. The conclusion highlights the potential of AI and machine learning in revolutionizing the health insurance industry by providing more accurate premium estimations, personalized insurance plans, and faster claim settlements. In the paper by Orji and Ukwandu (2024), the authors deployed three regression-based ensemble machine learning models: Extreme Gradient Boosting (XGBoost), Gradient-boosting Machine (GBM), and Random Forest (RF) for predicting medical insurance costs. Explainable AI methods (SHAP and ICE plots) are used to explain the key determinant factors influencing premium prices. The XGBoost model achieved better overall performance, while the RF model recorded a lesser prediction error and consumed fewer computing resources. The ICE plots provided more detailed interactions between variables compared to SHAP analysis. The study aims to assist policymakers, insurers, and potential medical insurance buyers in their decision-making process.

##### 3.1.2.2 AI for health insurance fraud detection:

The paper by Kapadiya et al. (2022) addresses the detection and prevention of healthcare insurance fraud using Artificial Intelligence (AI) and blockchain technology to mitigate financial losses and ensure trust among stakeholders. The paper utilizes AI algorithms, specifically machine learning models, to analyze health insurance data for identifying fraudulent activities, employing supervised learning methodologies to detect patterns and anomalies indicating potential fraud. The efficiency of the model is not explicitly mentioned. The main finding is the successful implementation of a blockchain and AI-based system for healthcare insurance fraud detection, which increases transparency and trust between insurance providers and subscribers. The conclusion highlights the potential of integrating wearable devices for real-time data collection and analysis in fraud detection systems while addressing challenges such as class imbalance, data standardization, and scalability.

##### 3.1.2.3 AI for health insurance data analysis and decision support:

The research by Thesmar et al. (2019) explores the potential benefits and challenges of integrating artificial intelligence (AI) with healthcare claims data. The paper discusses the potential of using machine learning algorithms to learn relationships from claims data and improve data analytics, enabling the extraction of valuable insights from large and complex datasets. The main finding is that AI, when combined with claims data, can help identify and reduce common biases in healthcare, detect complex patterns for early disease detection and underdiagnosed conditions, and ultimately lead to more informed decision-making in healthcare. The paper emphasizes considering patient confidentiality, methodological transparency, and potential discrimination when utilizing AI with claims data. The study by Isa et al. (2022) sheds light on the significant impact of AI on various aspects of health financing, including governance, revenue raising, pooling, and strategic purchasing. The paper explores how AI can assist in classifying beneficiaries of health insurance operators based on their financial sustainability, sociodemographic characteristics, and healthcare cost history. It also discusses the use of AI methodologies to design need-based and optimal insurance packages. The paper serves as a valuable resource for policymakers, healthcare professionals, and researchers interested in leveraging AI technologies to enhance health financing systems, optimize resource allocation, and improve decision-making processes in the context of health insurance. The paper by Ramezani et al. (2023) provides a comprehensive overview of how artificial intelligence (AI) techniques can be applied to various aspects of health financing, including health insurance. The paper discusses the potential of leveraging big data, machine learning, and AI capabilities to enhance premium modeling, risk pooling, strategic purchasing decisions, and customer-centric plan recommendations in health insurance programs and providers. The paper comprehensively maps the current and prospective applications of AI across all domains of health financing, with health insurance being an important area of focus. The paper Balboa et al. (2022) discusses the potential for AI to enhance the understanding of mental health risk factors and expand access to insurance for individuals with mental health conditions. The paper explores the integration of digital mental health tools and data from telehealth services into underwriting and pricing processes while considering data protection and consent considerations. The main finding is that improved data availability could open up additional products and underwriting designs, which could further expand access to insurance for those with mental health conditions. The paper emphasizes the importance of accuracy, data protection, and being mindful of data privacy rights when utilizing AI applications in health insurance.

##### 3.1.2.4 AI for health insurance explainability and interpretability:

The study by Bora et al. (2022) aims to provide explanations for the outcomes of complex Machine Learning algorithms used to predict the cost of health insurance. The paper utilizes Multiple Linear Regression and Random Forest algorithms for predicting insurance premium costs, followed by an explanation of the predicted results using Explainable AI (XAI) techniques like LIME and SHAP. The main finding is that these techniques can help check the correctness of prediction models, as domain experts can analyze the features that affect the outcome the most and provide their expertise. The conclusion aims to provide a better user experience and build trust between users and machine learning models by offering explanations for the predicted insurance premium costs.

#### 3.1.3 Property insurance applications

The reviewed literature highlights the versatility and transformative potential of AI and machine learning in the property, home, and casualty insurance industry, spanning risk assessment, fraud detection, home valuation, and operational optimization. The seamless integration of these advanced technologies has the power to drive innovation, improve customer experiences, and enhance the overall competitiveness and financial performance of insurance providers in the rapidly evolving landscape of the insurance sector. This review synthesizes the key contributions of the literature in showcasing the pivotal role of AI in revolutionizing various aspects of the insurance sector.

##### 3.1.3.1 Risk assessment and pricing

Several papers demonstrate the application of AI and machine learning techniques to enhance risk assessment and pricing in the property insurance domain. The paper by Rajagopal (2020) highlights how insurers can leverage Earth observation data and predictive models powered by AI to evaluate risks and improve decision-making processes dynamically. The author delves into how AI technologies, such as data analytics, predictive analytics, and machine learning, are being leveraged by Insurance Carriers to combat fraudulent claims, streamline the underwriting process, and optimize policy pricing. The efficiency of these AI models lies in their ability to automate claim processes, leading to faster claims settlements and improved customer satisfaction and operational efficiency. The study by Chen et al. (2022) explores the use of AI algorithms for the automated extraction of valuable information from building blueprints, enabling efficient and accurate risk assessment for industrial and commercial properties. The paper employs a combination of machine learning tools, including AI algorithms for preprocessing blueprints, identifying key features and objects, extracting textual information, and generating analytical outputs. While the efficiency metrics are not explicitly provided, the approach demonstrates the potential for rapid extraction of blueprint information, relieving risk engineers of routine tasks and allowing them to focus on higher-value assessments. Furthermore, the work by Dabbugudi (2022) showcases the implementation of AI models, such as neural networks, decision trees, and natural language processing, to streamline underwriting processes and enhance pricing models in the property and casualty insurance sector. The efficiency of these models is demonstrated through significant time reduction in building pricing frameworks, improved risk assessment accuracy, and enhanced customer satisfaction.

##### 3.1.3.2 Fraud detection

Addressing the challenge of fraudulent claims in property insurance, several papers have explored the application of machine learning techniques for improved fraud detection. The study by Severino and Peng (2021) utilizes ensemble-based classifiers, such as the random forest model, and deep neural networks to achieve superior performance in predicting fraud cases in residential and business insurance policies, outperforming traditional methods like logistic regression. Additionally, the work by Shi et al. (2016) proposes a copula regression model that effectively captures the complex dependence structure among different coverage types and over time, aids in the detection of fraudulent claims in property-casualty insurance. The efficiency of the copula model is demonstrated through estimation and inference based on the composite likelihood approach.

##### 3.1.3.3 Home valuation and pricing:

The accurate estimation of home prices is a crucial aspect of the property insurance industry, and several papers have addressed this challenge using advanced machine learning methodologies. Barr et al. (2017) present a gradient-boosted regression trees approach that outperforms traditional home price indices in capturing price dynamics at a granular level, showcasing the potential of AI-driven home valuation tools. Additionally, the work by Quijano Xacur and Garrido (2015) explores the use of generalized linear models (GLMs) with the Tweedie distribution to streamline the estimation of mean aggregate loss in property-casualty insurance. By fitting a GLM with a compound Poisson-gamma (CPG) distribution for the response, the Tweedie distribution allows for a more efficient estimation process, leading to more accurate and efficient premium estimations in the property and casualty insurance domain.

##### 3.1.3.4 Process optimization and customer experience

The integration of AI technologies has also been explored to drive digital transformation and optimize processes in the property and casualty insurance sector. The paper by Radu and Alexandru (2022) demonstrates the use of machine learning-powered virtual assistants to enhance customer service, reduce operational costs, and increase efficiency in property and home insurance operations. The efficiency of the model lies in its ability to learn and adapt to customer needs, improving response times and service quality. Similarly, the study by D'Arcy and Gorvett (2004a) showcases the application of dynamic financial analysis (DFA) and AI techniques to support strategic decision-making, such as determining optimal exposure growth rates to maximize long-term profitability in property-liability insurance. The efficiency of the model lies in its ability to systematically evaluate strategic alternatives and identify an optimal growth rate based on mean-variance analysis, stochastic dominance, and constraints on leverage.

### 3.2 Application of AI in insurance sectors—Industry-wide adoption

The insurance industry is increasingly adapting AI to enhance various aspects of their operations, ranging from underwriting and risk assessment to claims processing and customer service. By leveraging the power of AI, insurance companies aim to streamline processes, improve decision-making, and deliver personalized experiences to their customers.

In this section, we will explore how different companies are applying AI solutions across various types of insurance, including automotive insurance, property insurance (home, commercial, and rental), and health insurance as summarized in Table 2. The categorization will highlight the specific applications of AI within each insurance type.

**Table 2:** AI Applications in Insurance (Commercial Applications).

**Ref**.	**References**	**Services**	**Coverage & Stakeholders**
1	Afiniti (online)	Customer-agent pairing, personalized interactions	Auto, property, health; insurers, customers
2	Allstate (online)	Claims automation	Auto; insurers, customers
3	Arity (online)	Usage-based insurance, risk assessment, underwriting, fraud detection	Auto; auto insurers
4	Avaamo (online)	Conversational AI, advisory automation	Auto, property, health; insurers, customers
5	Bold Penguin (online)	Commercial insurance matching, risk assessment, automation	Property; agents, brokers, carriers, small businesses
6	CAPE Analytics (online)	Property intelligence, risk assessment, underwriting	Property; property insurers
7	Clearcover (online)	Pricing, claims processing, customer service	Auto; insurers, customers
8	Duck Creek (online)	Policy administration	Auto, property, health; insurers
9	Flyreel (online)	Property insurance underwriting	Property; property insurers
10	Gradient AI (online)	Risk assessment, underwriting, claims processing	Auto, property, health; insurers
11	Guevara (online)	Insurance brokerage	Auto, property, health; customers, insurers
12	H2O.ai (online)	Predictive analytics, fraud detection, personalization	Auto, property, health; insurers
13	Hi Marley (online)	Customer service, communication	Auto, property, health; insurers, customers
14	INSHUR (online)	Mobile automotive insurance for professional drivers	Auto; professional drivers
15	Insurify (online)	Insurance matching	Automotive, property; customers
16	Ladder (online)	Online life insurance	Health; customers
17	Lapetus Solutions (online)	Underwriting, claims processing, risk assessment, customer service	Auto, property, health; insurers
18	Lemonade (online)	Digital insurance platform, chatbots	Property, health; customers
19	Lexion Insurance (online)	Contract management	Auto, property, health; insurers
20	Metromile (online)	Pay-per-mile automotive insurance	Auto; customers
21	Nauto (online)	Driver safety system for commercial fleets	Auto; fleet operators, insurers
22	Nayya (online)	Health benefits selection	Health; employers, employees
23	Shift Technology (online)	Fraud detection, claims automation	Property, health; insurers
24	Slice (online)	On-demand insurance for sharing economy	Property; sharing economy customers
25	Snapsheet (online)	Claims processing automation	Auto, property, health; insurers
26	SprouttInsurance (online)	Personalized life insurance recommendations	Health; customers
27	Tractable AI (online)	Claims assessment automation	Auto, property, health; insurers
28	Trupanion (online)	Health (Pet)	Health; pet owners
29	Verisk (online)	Data analytics, risk assessment, decision support	Health, auto; insurers
30	WorkFusion (online)	Intelligent document processing, automation	Auto, property, health; insurers
31	Yembo (online)	Virtual property analyses	Property; property insurers
32	ZestFinance (online)	Underwriting, risk assessment	Auto, property, health; P&C insurers
33	Zebra (online)	Insurance comparison	Auto; customers

#### 3.2.1 Automotive insurance

Arity (online) provides telematics and mobility data solutions for automotive insurance companies, aiding in pricing, marketing, and claims management through AI-driven analysis of driving behavior data. (Nauto, online) focuses on commercial fleet insurance, leveraging AI to analyze driving behavior, identify risks, and improve safety for fleet operators. Metromile (online) offers pay-per-mile automotive insurance, utilizing AI for personalized risk assessment, claims processing, and customer service based on individual driving patterns. Insurify (online) utilizes AI algorithms to match customers with suitable automotive insurance policies based on their specific needs and preferences. INSHUR (online) Provides mobile-first commercial automotive insurance solutions for rideshare drivers, likely using AI for risk assessment, claims processing, and customer engagement.

#### 3.2.2 Property insurance

CAPE Analytics (online) leverages AI and computer vision for accurate property risk assessment and underwriting in home and commercial property insurance. Lexion Insurance (online) AI-driven solution for property insurance underwriting, property inspection workflows, and risk management. Yembo (online) offers AI-powered virtual property analysis for claims assessment, risk evaluation, and property inspection in property insurance. Bold Penguin (online) utilizes AI algorithms for matching businesses with suitable insurance options, streamlining underwriting processes, and enabling personalized solutions across various insurance types, including small commercial insurance. Lemonade (online) Utilizes AI for underwriting, claims processing, and customer service in renters and homeowners insurance. Slice (online) Specializes in providing on-demand property insurance solutions for the sharing economy, such as home-sharing and rental insurance, likely using AI for underwriting and claims management.

#### 3.2.3 Health insurance

AI-driven underwriting, claims processing, fraud detection, and personalized health plan recommendations for individuals and employers. While the information is not explicitly stated, Allstate (online) may offer health insurance products through partnerships or subsidiaries, potentially utilizing AI for tasks like claims processing and customer service automation. Nayya (online) Utilizes AI to help employers choose suitable health insurance and employee benefits plans based on employee needs and preferences. Gradient AI (online) Offers AI solutions for insurance companies, including enhancing underwriting, claims processing, and fraud detection in the group health insurance sector. Ladder (online), an online life insurance company that may leverage AI for underwriting, customer service automation, and personalized policy recommendations. SprouttInsurance (online) employs AI to match individuals with suitable life insurance plans based on their unique circumstances and personal factors. Trupanion (online) offers pet insurance services, potentially benefiting from AI solutions for tasks like claims processing, risk assessment, and personalized policy recommendations, as other insurance companies employ.

The following insurance companies deploy AI to serve all three insurance types. Tractable AI (online) offers AI solutions for automating claims processing across auto, property, and health insurance. Avaamo (online) provides generative AI solutions and conversational AI for insurance aggregators, automotive insurance, property & casualty insurance, and life insurance. Gradient AI (online) offers AI solutions for insurance companies to enhance underwriting, claims processing, and fraud detection across various insurance sectors, including business owners, commercial auto, and group health. H2O.ai (online) provides AI and machine learning solutions for insurance companies to optimize underwriting, risk assessment, customer service, and other processes across different types of insurance, including health, automotive, property, and life insurance. Lapetus Solutions (online) leverages AI for underwriting, claims processing, risk assessment, and customer service in various insurance domains. Lexion Insurance (online) specializes in AI-powered contract management, including insurance contracts, applicable across various types of insurance. LexisNexis Risk Solutions conducted a study on Advanced Driver Assistance Systems (ADAS) and their impact on automotive insurance claims ADAS Analysis from LexisNexis Risk Solutions Sets Record Straight on U.S. Auto Claims Severity, (online). The analysis revealed that vehicles equipped with ADAS features showed significant reductions in loss costs: 23% for Bodily Injury, 14% for Property Damage, and 8% for Collision claims. These findings challenge the common belief that ADAS repair costs offset their benefits. The study suggests that insurers can use this data to offer more accurate policy discounts based on specific ADAS features present in vehicles. LexisNexis now offers VIN-level ADAS feature information through their Vehicle Build product, allowing insurers to better assess risk and meet customer expectations for lower rates on safer vehicles. ZestFinance (online) utilizes machine learning for underwriting and risk assessment in property and casualty insurance, including homeowners, renters, auto, and commercial property insurance.

### 3.3 Key considerations for AI adaptation in insurance

Although AI has the potential to revolutionize risk assessment, fraud detection, and customer service in the digital insurance landscape, it also encounters significant bottlenecks (Łyskawa et al., 2019). The Following key considerations must be addressed to ensure the successful and responsible deployment of AI in the insurance sector:

1. **Data quality and governance**: High-quality data is required to train an AI model effectively (Sambasivan et al., 2021; Whang et al., 2023). At the same time, data governance helps ensure AI model has the right information to work with accuracy, consistency, and security (Janssen et al., 2020; Liang et al., 2022). The insurance sector is not an exception. In particular, data governance practices are critical for AI applications in health insurance to protect sensitive health information, comply with regulations like the Health Insurance Portability and Accountability Act of 1996 (HIPAA) in the United States, and maintain consumer trust (Arigbabu et al., 2024).
2. **Ethical considerations**: Insurers must ensure ethical considerations (that includes fairness, transparency, and accountability in AI algorithms) before deploying AI in insurance processes to prevent biases and discrimination (Pisoni and Dìaz-Rodrìguez, 2023; Prince and Taylor, 2023). Mullins et al. (2021) introduced a hierarchical model for the European Insurance and Occupational Pensions Authority (EIOPA) to analyze ethical issues arising from the application of AI in various aspects of the European insurance market. Recently, Luxembourg Institute of Science & Technology (LIST) introduced a leaderboard (https://ai-sandbox.list.lu/) to tackle the ethical considerations of AI systems (Gomez-Vazquez et al., 2024). The leaderboard comprehensively assesses and benchmarks Large Language Models (LLMs) based on a set of ethical biases. It also evaluates LLMs across seven ethical biases, including ageism, LGBTIQ+phobia, political bias, racism, religious bias, sexism, and xenophobia. Therefore, The leaderboard can contribute to addressing the ethical considerations associated with AI deployments in the insurance sector.
3. **Regulatory compliance**: Following the privacy and security regulations is essential for AI applications in the insurance sector (Feetham and Amos, 2012) Kandepu (2023). Insurers need to navigate complex regulatory requirements (Kochenburger and Salve, 2023) and data protection laws in different jurisdictions when implementing AI solutions. A few such EU regulations and listed in Table 3 where the European Insurance and Occupational Pensions Authority (EIOPA) plays a significant role in supporting their effective implementation throughout the European Union.
4. **Interpretability and explainability**: To build trust with the end users and stakeholders, there is an imperative need for transparent and interpretable AI models. Explainable AI (XAI) techniques can be used to provide insights into the decision-making process of AI algorithms (Owens et al., 2022b,a) to drive the future of insurance practices toward a more ethical and trustful direction. In this direction, Koster et al. (2021) developed a checklist for insurance companies to implement XAI techniques and build trust with stakeholders. Gramegna and Giudici (2020) propose an XAI approach that leverages Shapley values from an XGBoost classifier. This allows insurers to understand the key factors influencing customer decisions in real time. A recent study by McDonnell et al. (2023) explored TabNet, a deep learning architecture for claim prediction using telematics data. They demonstrate that TabNet performs better than traditional models like XGBoost and Logistic Regression in both accuracy and interoperability. As previously described, LIST's leaderboard (https://ai-sandbox.list.lu/) emphasizes the importance of explainability in the evaluation process. The leaderboard offers a detailed report explaining the tests conducted and the evaluation criteria, promoting transparency and interpretability of the assessment process.
5. **Innovation, continuous learning, and adaptation**: From risk assessment to product development, the insurance sector is undergoing a revolution driven by constant innovation. In an ever-shifting risk landscape, insurers should ensure continuous retraining of AI models on dynamic risk factors and fraud patterns to stay relevant and accurate over time (Grossberg, 2020). This requires a commitment toward continuous learning and adaptation to cope with changing market conditions and customer needs Dhieb et al. (2020). LIST's leaderboard (https://ai-sandbox.list.lu/), as described in the paper Gomez-Vazquez et al. (2024), allows for continuous learning and adaptation to enable the integration of new LLMs and test cases. LLMs can analyze massive amounts of data, including customer information, past claims, and market trends (Balona, 2023). Using the capabilities of LLMs, insurers can gain a deeper understanding of their customers' portfolios which helps insurers to offer personalized premiums that better reflect individual circumstances (Dimri et al., 2019). While LLMs hold immense promise for the insurance sector, there are challenges to consider, such as data bias, explainability, security concerns, and most importantly LLM hallucination problem (Qiu et al., 2023). This is where Retrieval-Augmented Generation (RAG) comes into play (Lewis et al., 2020). RAG offers a potential solution by combining the strengths of LLMs with retrieval-based approaches (Fatehkia et al., 2024), chhikara2024few. The insurance sector must position itself as a more efficient, data-driven, and customer-centric industry by collaborating with technology partners and industry experts.
6. **Cultural/societal acceptance**: The effect of digitalization in the insurance sector can face hurdles due to societal and cultural reservations (Burchardt and Maisch, 2019). There can be trust issues as people may be doubtful of the decision-making algorithms for policy pricing or claims. The human touch in such sensitive matters would be desirable. Participation from certain demographics in a fully digitalized insurance can be excluded due to the lack of comfort/ confidence with online interactions and technological illiteracy (Butt et al., 2023; Fernando and Jain, 2022; Leal-Rodrìguez et al., 2023). Insurers need to foster a culture of innovation and collaboration to drive the adoption of AI technologies across different insurance domains (Kulkova, 2021).

**Table 3:** Insurance Regulations in Europe.

**References**	**Year**	**Description**
PRIIP (2014)	2014	A regulation that aims to improve the transparency and comparability of packaged retail and insurance-based investment products (PRIIPs) across the European Union.
Solvency II (2016)	2016	A set of regulatory requirements aimed at harmonizing insurance regulation across the European Union, focusing on risk management and capital requirements to ensure they can meet policyholder obligations.
(GDPR)	2016	Not specific to insurance, but crucial for data privacy. A regulation on data protection and privacy for individuals within the European Union and the European Economic Area. It addresses the transfer of personal data outside the EU and EEA areas.
(IDD)	2016	A European Union directive that aims to create a level playing field for all participants involved in the selling of insurance products. It enhances consumer protection and harmonizes insurance distribution rules across the EU.
EU Act (2023)	2023	The European Union's AI Act will significantly impact the insurance industry. It introduces stricter oversight on AI-driven processes in insurance. These include risk assessment, pricing, and claims handling. Insurers must categorize their AI systems based on risk levels. High-risk systems will face more rigorous requirements.

By addressing these key challenges and adopting the transformative potential of artificial intelligence, insurance companies can position themselves for success in the rapidly evolving landscape of the insurance sector.

### 3.4 Hypotheses development

This section consolidates academic research, industry advancements, and sustainability-oriented regulatory considerations in AI-driven insurance. The development of hypotheses aims to bridge gaps between technological innovation, ethical governance, and environmental/social responsibility.

#### 3.4.1 AI applications in insurance: domain-specific insights

This subsection analyzes AI's role in automotive, health, and property insurance through sustainability and regulatory lenses, highlighting industry-specific advancements and challenges. The following tables present a structured analysis of domain-specific AI implementations and their corresponding sustainability and regulatory dimensions.

##### 3.4.1.1 Automotive insurance domain

The automotive insurance sector exhibits pioneering applications of AI in risk assessment and claims automation. Table 4 demonstrates how telematics and computer vision technologies are reshaping traditional automotive insurance models while addressing sustainability objectives. The integration of environmental parameters into risk calculations represents a significant shift toward climate-conscious insurance practices.

**Table 4:** Domain analysis: automotive insurance.

**Automotive insurance**	
AI Applications	• **Telematics and usage-based insurance (UBI):** AI-driven telematics assess driver behavior and contextual risks Masello et al. (2023). Machine learning models enhance dynamic pricing models using real-time environmental data. • **Automated Damage Detection:** CNNs and deep learning facilitate automated claims processing?.
Sustainability Integration	• **Climate resilience:** AI models integrating environmental data Mahadik et al. (2025) enable climate-risk pricing. • **Eco-friendly incentives:** UBI encourages low-emission driving, promoting sustainability goals.
Regulatory Alignment	• **EU AI act compliance:** Transparent AI-driven UBI systems prevent “high-risk” categorization under EU regulations EU Act (2023).
**Hypothesis 1:** *AI-driven environmental data integration and ESG metrics enhance automotive insurance risk accuracy and promote policyholder sustainability behaviors*.	

##### 3.4.1.2 Health insurance domain

In health insurance, AI applications exhibit dual functionality in cost prediction and fraud prevention. Table 5 illustrates how these technologies extend beyond operational efficiency to address preventive healthcare and inclusive coverage–critical sustainability objectives. Explainable AI emerges as a key enabler for regulatory compliance while maintaining algorithmic transparency.

**Table 5:** Domain Analysis: Health Insurance.

**Health insurance**	
AI Applications	• **Premium prediction:** Deep learning models predict healthcare costs based on lifestyle data Samiuddin et al. (2023). • **Fraud detection:** Blockchain-AI integration improves claims verification Kapadiya et al. (2022).
Sustainability Integration	• **Preventive care:** AI-driven wellness programs encourage sustainable nutrition and health practices Van Erp et al. (2021). • **Mental health assessment:** AI supports inclusive insurance policies through risk assessment Balasubramanian et al. (2018).
Regulatory Alignment	• **GDPR & HIPAA compliance:** Explainable AI (XAI) ensures transparency in health data usage Bora et al. (2022).
**Hypothesis 2:** *AI-driven preventive healthcare interventions and XAI frameworks enhance health insurance sustainability while ensuring regulatory compliance*.	

##### 3.4.1.3 Property insurance domain

Property insurance represents a domain where climate concerns directly intersect with risk management. Table 6 synthesizes how AI enables advanced climate risk modeling and parametric insurance solutions. The integration of satellite imagery and IoT technologies exemplifies how technological innovation can address evolving climate challenges while meeting regulatory disclosure requirements.

**Table 6:** Domain Analysis: Property Insurance.

**Property insurance**	
AI applications	• **Risk assessment:** AI utilizes satellite imagery and computer vision for climate risk evaluation (Chen et al., 2022). • **Fraud detection:** Machine learning classifiers enhance fraud identification (Severino and Peng, 2021).
Sustainability integration	• **Climate adaptation:** Bayesian models support parametric insurance for sustainable farming (Ali, 2024). • **Smart grid sensors:** IoT-enabled AI assesses infrastructure risks in climate resilience initiatives (Mahadik et al., 2025).
Regulatory alignment	• **Climate risk disclosure:** AI ensures compliance with TCFD guidelines for transparent risk disclosure.
**Hypothesis 3:** *AI-enabled climate risk assessment and ESG metrics improve regulatory adherence and sustainability outcomes in property insurance*.	

#### 3.4.2 Cross-domain synergies: sustainability and regulation

Beyond domain-specific applications, this analysis identifies transdisciplinary patterns in AI-driven insurance. Table 7 synthesizes emergent themes across insurance domains, revealing how ESG imperatives and regulatory frameworks transcend sectoral boundaries. The findings suggest that AI governance frameworks integrating ESG considerations represent an essential pathway toward sustainable insurance innovation.

**Table 7:** Cross-domain analysis and final hypothesis.

**Cross-domain synergies**	
Emerging themes	• **ESG-driven innovation:** Insurers with strong ESG alignment integrate AI for ethical risk governance (Habib and Mourad, 2024). • **Co-creation models:** Stakeholder-inclusive AI design fosters sustainable decision-making (Ali, 2024).
Regulatory compliance	• **Dynamic governance:** AI must adapt to evolving regulatory landscapes (EU Act, 2023). • **Bias mitigation:** Explainable AI techniques mitigate algorithmic discrimination in risk assessment (Mullins et al., 2021).
**Hypothesis 4:** *Insurers integrating AI within ESG and regulatory frameworks achieve greater trust, compliance, and resilience*.	

## 4 Methodology

This study employs a systematic literature review to bridge academic research and industry practices in AI-driven insurance. Aligning with PRISMA guidelines (Page et al., 2021), we analyze AI applications across automotive, health, and property insurance domains, focusing on operational efficiency, ethical alignment, and regulatory compliance.

### 4.1 Research context

The insurance industry is undergoing a significant transformation driven by advancements in artificial intelligence (AI). This technology is being applied across various domains, including automotive, health, and property insurance, to enhance efficiency, improve risk assessment, and drive innovation. However, the integration of AI in insurance processes presents several challenges, such as data quality, ethical considerations, regulatory compliance, and the need for explainable AI models. This systematic review aims to provide a comprehensive overview of the current state of AI applications in the insurance industry, focusing on recent research contributions, industry adoption trends, and key challenges.

To guide this systematic review, the following research questions were formulated:

Q1: *What is the current state of research and industry adoption of artificial intelligence (AI) techniques in various insurance domains, including automotive/vehicle, health, property, and other types of insurance?*

This broad question can be further divided into the following sub-questions:

Q1.1: *What are the recent research contributions exploring the application of AI techniques in automotive/vehicle, health, and property insurance domains, and what specific AI models or algorithms have been utilized for tasks such as risk assessment, fraud detection, claims processing, and customer service?*

Q1.2: *Which companies are actively adopting AI solutions across various insurance domains (automotive/vehicle, health, property, and others), and what specific AI applications or services are they offering in areas like underwriting, risk assessment, claims processing, fraud detection, and customer service?*

Q1.3: *What are the key challenges and considerations associated with integrating AI in the insurance sector, including data quality and governance, ethical considerations, regulatory compliance, interpretability and explainability, innovation and adaptation, and societal/cultural acceptance?*

### 4.2 Research design

To address the research questions and objectives, we employed a systematic review methodology following the PRISMA guidelines. This approach ensures a thorough and unbiased evaluation of the literature, providing a comprehensive understanding of AI applications in the insurance sector. The methodology follows a three-phase structure:

#### 4.2.1 Data collection

##### 4.2.1.1 Data source

To identify relevant studies for this systematic review, we conducted a comprehensive search (besides Google) in the following electronic databases for papers and companies:

1. Google Scholar (https://scholar.google.com/)
2. dblp: computer science bibliography (https://dblp.org)
3. Web of Science (https://www.webofscience.com/wos/woscc/basic-search)
4. linkedin (https://www.linkedin.com/), specially for companies.
5. Google Patents(https://patents.google.com/).

##### 4.2.1.2 Search strategy

The search strategy included a combination of keywords and Boolean operators (AND, OR, -) related to the main concepts of the review, such as artificial intelligence, deep learning, machine learning, insurance, automotive, health, property, regulation, Impact of AI, companies, firms and their synonyms and variations. Additionally, we performed manual searches in the reference lists of relevant studies and consulted subject-matter experts to identify any potentially missed publications. Consider the sample search strategy that aims to find articles that explore the application of various AI techniques in automotive insurance domains with a focus on original research rather than general overviews.: (AI OR “artificial intelligence” OR “machine learning” OR“deep learning”) AND (insurance OR insurer OR underwriting) AND(automotive OR “vehicle insurance”)-general -overview -survey -review. The search uses parentheses to group related concepts. All these groups are then connected with the AND operator. The exclusion terms (prefix “-”) exclude studies focused on general applications of AI in the insurance industry, general overviews of AI in insurance, and broad survey and review papers.

#### 4.2.2 Screening process

##### 4.2.2.1 Inclusion and exclusion criteria

The inclusion criteria (Table 8) ensure that the selected papers and companies are relevant to the focus of the systematic review, which is the application of AI in various insurance domains. The exclusion criteria help filter out companies or papers that do not meet the specific criteria or lack sufficient information about their AI-based insurance offerings.

**Table 8:** Inclusion and exclusion criteria for scientific papers and companies.

**Inclusion criteria** ^*****a*****^		**Exclusion criteria** ^*****b*****^	
**Academic papers**	**Companies**	**Academic papers**	**Companies**
1. Studies focusing on AI techniques in specific insurance domains (automotive, health, property).	1. Companies offer AI-based automotive, health, or property insurance solutions.	1. Studies that do not directly address AI use in specific insurance domains.	1. Companies that do not offer AI-based solutions for specific insurance domains.
2. Studies in peer-reviewed journals, conferences, research reports, and reviews.	2. Companies providing detailed information on AI applications via reports and patents.	2. Opinion pieces, editorials, or reviews without empirical data.	2. Companies whose AI offerings are not explicitly related to target domains.
3. Studies written in English.	3. Companies with dedicated website showing AI-based insurance offerings.	3. Duplicates or studies reporting the same data.	3. Companies without clear information on their AI-based insurance solutions.
4. Papers published between 2015-2024.		4. Studies on general insurance without domain insights.	

^a^ Criteria for selecting relevant scientific papers and companies for inclusion in the study. ^b^ Criteria for excluding scientific papers and companies from the study. *Source*: Compiled based on research objectives and scope of the study.

##### 4.2.2.2 Two-Phase Screening

The screening process was conducted using a systematic two-phase approach, evaluating both academic publications and industry implementations of AI applications in the insurance domain. This comprehensive review process was structured to capture the state-of-the-art research and practical applications across six key insurance service classes:

1. Pricing and risk assessment.
2. Claims automation and damage detection.
3. Customer personalization and premium prediction.
4. Policyholder behavior analysis.
5. Strategic decision-making and process optimization.
6. Fraud detection.

In the initial phase, two independent reviewers (authors, A and B) evaluated documents from two primary sources mentioned in Table 8, Inclusion Criteria column (point 2 for both scientific papers and companies, respectively) following the search strategy mentioned in Section 4.2.1. Inter-reviewer discrepancies were systematically resolved through consensus discussions, with unresolved cases adjudicated by the other two reviewers (authors, C and D).

The full-text assessment phase involved the same two reviewers (A and B) independently examining the complete documents. The academic literature was assessed based on theoretical foundation, methodology, AI techniques employed, empirical validation, insurance sector coverage, practical implications, and ethical considerations. The industry implementations were evaluated on parameters like technology readiness level, scale of deployment, performance metrics, market impact, regulatory compliance, and implementation challenges. All exclusion decisions during the full-text review phase were documented with specific rationales, ensuring a comprehensive representation of both theoretical advances and practical applications of AI in the insurance industry. This dual-perspective approach, combining academic research and industry implementations, enabled a more complete understanding of the current state of AI applications in insurance, highlighting not only research gaps but also the practical challenges and successes in the real-world deployment of AI-driven solutions across the six key service classes. The final refined version of the analysis is shown in Tables 9, 10.

**Table 9:** Classification of AI applications in insurance literature by type and category.

**Service class**	**Automotive**	**Health**	**Property**
Pricing and risk assessment	(Ayuso Mercedes, 2019; Gao et al., 2022; Reimers and Shiller, 2018; Huang and Meng, 2019; Mullins et al., 2021)	(Samiuddin et al., 2023; Thesmar et al., 2019; Balboa et al., 2022; Mullins et al., 2021; Orji and Ukwandu, 2024)	(Rajagopal, 2020; Chen et al., 2022; Dabbugudi, 2022; Quijano Xacur and Garrido, 2015; Mullins et al., 2021)
Claims automation and damage detection	(Rababaah, 2023; Meenakshi and Sivasubramanian, 2023; Fouad et al., 2023; Samarasinghe et al., 2021; Sree et al., 2023)	(Mahohoho et al., 2023)	(Mahohoho et al., 2023)
Customer personalization and premium prediction	(Sinha et al., 2021; Nanda et al., 2023)	(Kaushik et al., 2022; Orji and Ukwandu, 2024; Bora et al., 2022)	(Barr et al., 2017)
Policyholder behavior analysis	(Guillen et al., 2021; Li et al., 2023; Ma et al., 2018; Masello et al., 2023)	(Isa et al., 2022; Ramezani et al., 2023; Thesmar et al., 2019)	-
Strategic decision-making and process optimization	(Mullins et al., 2021)	(Isa et al., 2022; Ramezani et al., 2023; Mullins et al., 2021)	(Radu and Alexandru, 2022; D'Arcy and Gorvett, 2004a; Mullins et al., 2021)
Fraud detection	(Benedek and Nagy, 2023)	(Kapadiya et al., 2022)	(Severino and Peng, 2021; Shi et al., 2016)

**Table 10:** Industry applications distribution across insurance domains.

**Service class**	**Automotive**	**Property**	**Health**
Pricing and risk assessment	(CAPE Analytics, online; Flyreel, online; Gradient AI, online; Yembo, online; ZestFinance, online)	(Gradient AI, online; Lapetus Solutions, online; ZestFinance, online)	(Arity, online; Clearcover, online; Gradient AI, online; Metromile, online; ZestFinance, online)
Claims automation and damage detection	(Snapsheet, online; Tractable AI, online; WorkFusion, online)	(Lapetus Solutions, online; Snapsheet, online; Tractable AI, online; WorkFusion, online)	(Allstate, online; Snapsheet, online; Tractable AI, online; WorkFusion, online)
Customer personalization and premium prediction	(Bold Penguin, online; Lemonade, online; Slice, online)	(Ladder, online; Nayya, online; SprouttInsurance, online; Trupanion, online)	(Afiniti, online; INSHUR, online; Insurify, online; Zebra, online)
Policyholder behavior analysis	(Avaamo, online; Hi Marley, online; Lexion Insurance, online)	(Avaamo, online; Hi Marley, online; Lexion Insurance, online)	(Avaamo, online; Hi Marley, online; Lexion Insurance, online; Nauto, online)
Strategic decision-making and process optimization	(Duck Creek, online; Guevara, online; H2O.ai, online)	(Duck Creek, online; Guevara, online; H2O.ai, online)	(Duck Creek, online; Guevara, online; H2O.ai, online)
Fraud detection	(H2O.ai, online; Shift Technology, online)	(H2O.ai, online; Shift Technology, online; Verisk, online)	(Arity, online; H2O.ai, online; Verisk, online)

#### 4.2.3 Data extraction

To address our research questions, we extracted the following data from the search results:

- For research question Q1.1 (reflected in Section 3.1): Author(s), Key Findings (e.g. contribution in risk assessment, fraud detection, etc.), Methodology (AI model/algorithm used—CNN, DNN, etc.), Coverage (e.g. insurance type- automotive, health, property), and Stackholders (e.g., Target audience- underwriters, insurers, clients, etc.)
- For research question Q1.2 (reflected in Section 3.2): Company name, Services, Coverage, and Stakeholders.
- For research question Q1.3 (reflected in Section 3.3) Data quality and governance, ethical considerations, Regulatory compliance, Explainability, Adaptation, Societal acceptance.

This data extraction process ensures we capture the most relevant information to address our research questions and provide a comprehensive overview of AI applications in specific insurance domains.

### 4.3 Analysis procedures

**Figure 1:**
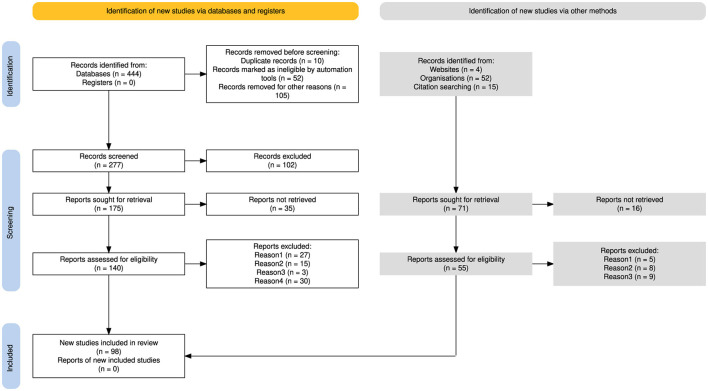
PRISMA diagram for the literature review.

The PRISMA flowchart (Haddaway et al., 2022) in Figure 1 provides a transparent illustration of the literature search and selection process undertaken in this paper. This diagram is generated using the online tool available at https://estech.shinyapps.io/prisma_flowdiagram/. The initial search yielded a total of 444 papers from various databases mentioned in Section 4.2.1. We use Zotero (https://www.zotero.org/) to identify and remove 52 duplicate papers. In addition, 10 papers were excluded based on manual screening for duplication. 105 papers were excluded for various reasons, e.g. language restriction, publication date, journal type, missing citation, etc. This resulted in a pool of 277 papers for further evaluation. Following a detailed review of abstracts, 102 papers did not meet the inclusion criteria. This left 175 potentially relevant studies. However, full-text access could not be obtained for 35 of these articles, resulting in 140 full-text articles for further in-depth analysis. In the next step, we removed a total of 75 papers based on the exclusion criteria provided in Table 8. Following this analysis, 65 papers were identified as meeting all inclusion criteria and were ultimately included in the review. Our search extended beyond academic publications to incorporate industry perspectives. Four relevant blogs were identified, which led to the exploration of 52 company websites. Additionally, reference lists of included studies yielded 15 further potential sources. From these 71 additional sources, 16 were inaccessible. After a detailed review of the remaining 55 sources, exclusions were made based on the criteria mentioned in Table 8. 22 websites did not meet the inclusion criteria, leading to a refined set of 33 potential studies for subsequent analysis.

By following this structured methodology, this review aims to provide valuable insights into the current state and future directions of AI applications in the insurance industry, highlighting research gaps and practical challenges.

## 5 Significant findings

**Figure 2:**
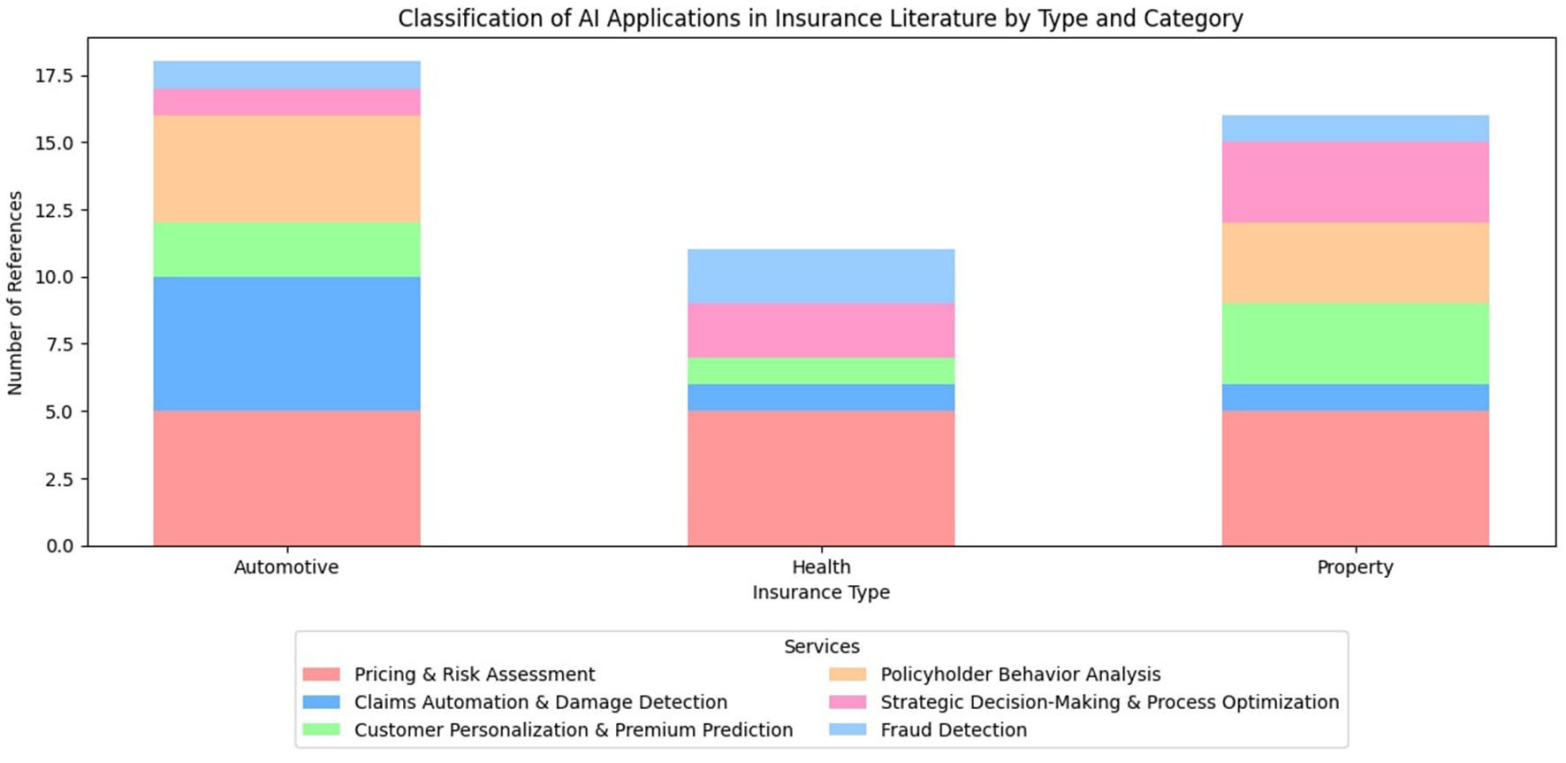
Classification of AI applications in insurance literature by type and category.

**Figure 3:**
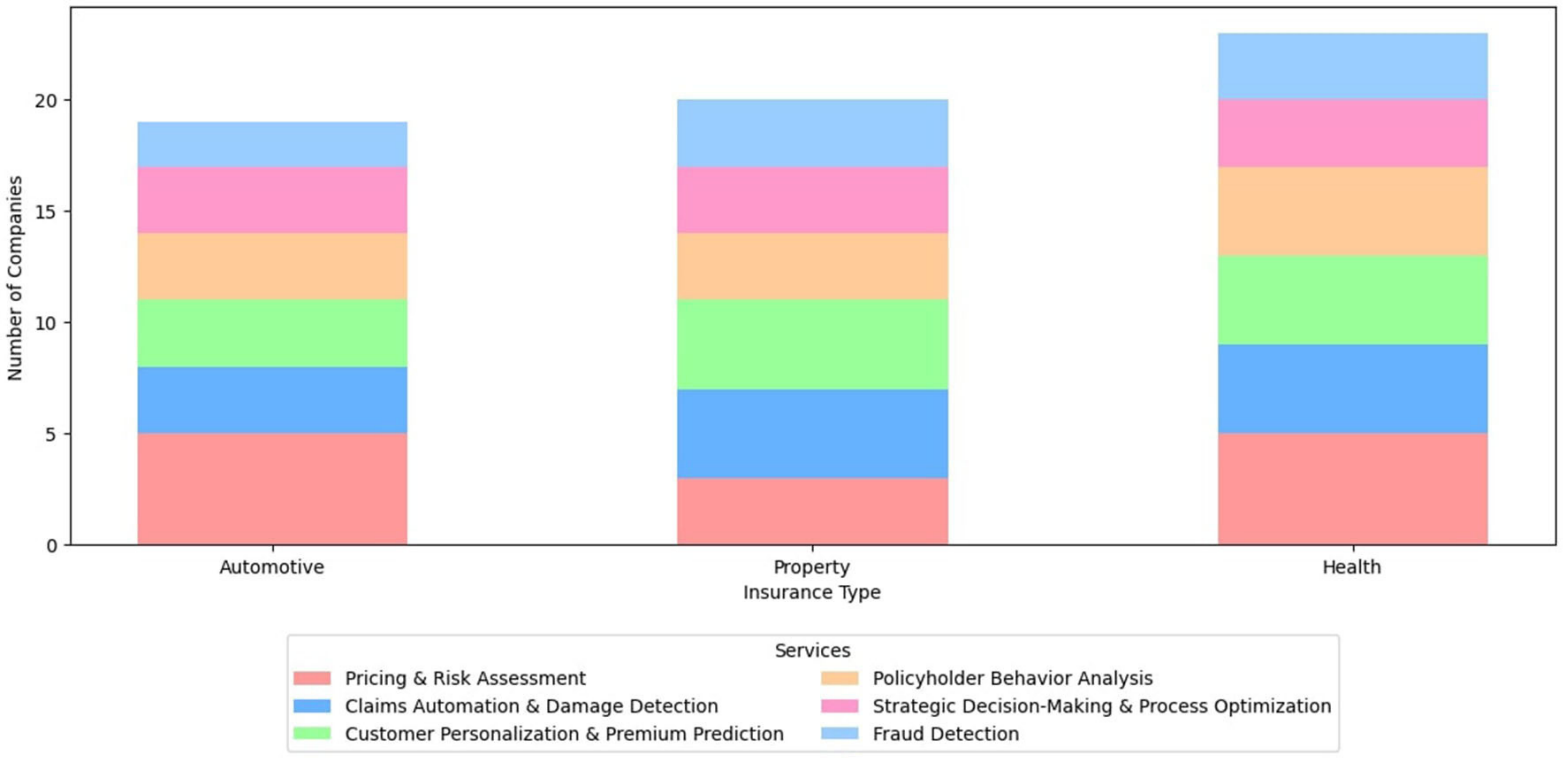
Classification of AI applications in insurance industry by type and category.

The stack bar graphs presented in Figures 2, 3 are derived from the data in Tables 9, 10, respectively. Both Tables 9, 10 in turn were infered from the original data in Tables 9, 10, respectively. The screening criteria outlined in Section 4 were applied to filter and refine the data before generating the visualization. Therefore, both the tables classify the AI Applications in the insurance industry and the academic literature by insurance types.

### 5.1 Academic literature

Our analysis of 36 research papers across three insurance domains and six service categories reveals significant trends in AI applications for insurance in Figure 2. Pricing and Risk Assessment dominates with 42% of papers, highlighting its industry priority. Auto insurance leads research focus (50% of papers), particularly in damage detection and claims automation. Health insurance follows (44%), with balanced coverage across categories except claims automation. Property insurance (33%) shows notable gaps in policyholder behavior analysis. The research landscape demonstrates an increasing focus on explainable AI (Mullins et al., 2021; Bora et al., 2022) and real-time data integration, especially in auto insurance applications. Claims automation and damage detection remain heavily concentrated in auto insurance (5 out of 7 papers), while fraud detection receives relatively balanced but limited attention across all insurance types. Some papers, particularly those addressing strategic decision-making and price modeling, span multiple insurance types, indicating a trend toward integrated approaches. Notable research gaps exist in property insurance policyholder behavior analysis and claims automation for health and property sectors, suggesting opportunities for future research directions.

### 5.2 Industry applications

The distribution of applications across insurance domains reveals several significant patterns in Figure 3. Health insurance leads with 23 implementations, followed by Property (20) and Automotive (19) domains. The highest concentration appears in Pricing & Risk Assessment, particularly in Health and Automotive sectors (5 implementations each). Claims Automation maintains consistent adoption (3–4 implementations) across all domains, reflecting its industry-wide importance. Notably, Customer Personalization shows stronger presence in Health and Property domains (4 each) compared to Automotive (3), suggesting varying complexity in customer segmentation needs. Strategic Decision-Making demonstrates uniform adoption (3 implementations) across all domains, indicating similar organizational requirements regardless of insurance type. Several companies, such as H2O.ai (online) and Duck Creek (online), operate across multiple domains, highlighting the emergence of domain-agnostic solutions. The narrow range of implementations (2–5) per category suggests a balanced approach to service adoption rather than concentrated investment in specific areas. This distribution pattern indicates that insurance sectors have reached comparable levels of technological maturity while maintaining domain-specific focus where necessary.

### 5.3 Comparison

Our analysis reveals striking differences between academic research and industry implementation of AI in insurance. Academic research strongly favors Pricing & Risk Assessment. This category alone accounts for 42% of all papers we analyzed. The industry, however, tells a different story. We found a more evenly distributed adoption of AI services. Health insurance leads with 23 implementations. Property and Automotive sectors follow closely with 20 and 19 implementations respectively.

Through our analysis, we identified a few critical research gaps. Property insurance lacks academic attention in both policyholder behavior analysis and claims automation. Industry has implemented these solutions, but research lags behind. The strategic decision-making shows consistent industry adoption with 3 implementations per domain. Academic coverage varies widely here—1 paper for automotive, 2 for property, and 3 for health insurance. Through our analysis, we identified three critical research gaps. First, property insurance lacks academic attention in both policyholder behavior analysis and claims automation. Industry has implemented these solutions, but research lags behind. Second, strategic decision-making shows consistent industry adoption with 3 implementations per domain. Academic coverage varies widely here—1 paper for automotive, 2 for property, and 3 for health insurance. Fraud detection receives minimal attention across both academia and industry. We found only 1–3 implementations or papers per domain.

## 6 Discussion

This section critically examines the findings of this study in relation to existing theoretical frameworks and literature. This section is divided into three subsections: Theoretical Implications, Managerial/Policy Implications, and Limitations and Future Research Agenda.

### 6.1 Theoretical implications

Our study provides a comprehensive review of AI applications in the insurance industry, focusing on automotive, health, and property insurance domains. By employing the PRISMA methodology, we systematically analyzed recent academic research and industry practices. Our findings align with and extend several theoretical frameworks discussed in the literature.

The study confirms that perceived usefulness (PU) and perceived ease of use (PEU) significantly influence AI adoption in insurance. However, contrary to classical TAM assumptions (Davis et al., 1989), regulatory constraints and ethical considerations (e.g., algorithmic bias) emerge as additional determinants of acceptance. Our study extends TAM by highlighting the unique challenges of deploying AI in risk-sensitive environments, aligning with recent research by Gabelaia et al. (2024). Our findings resonate with the Diffusion of Innovation Theory by Rogers et al. (2014), which explains the uneven adoption of AI across different market segments and regions. The integration of AI in insurance risk management aligns with the Expected Utility Theory (Von Neumann and Morgenstern, 2007). AI systems enhance traditional probabilistic risk evaluation by identifying subtle patterns in large datasets, thereby improving the accuracy of risk assessments. Prospect Theory by Kahneman and Tversky (2013) helps explain the human dimensions of risk assessment and decision-making in insurance. Our study highlights the importance of acknowledging the heuristic nature of human decision-making when integrating AI, as suggested by Gigerenzer and Brighton (Gigerenzer and Brighton, 2009). Lastly, the Corporate Social Responsibility (CSR) framework by Carroll (2016) provides a valuable lens for examining ethical AI implementation in insurance. Our findings emphasize the need for responsible AI deployment that balances profit motives with fairness, accountability, and transparency, as argued by Keller (2020).

### 6.2 Managerial/policy implications

Our study offers several managerial and policy implications for the insurance industry. By providing a comprehensive analysis of AI applications, we identify key challenges and opportunities for insurers and policymakers. For insurers, our findings highlight the importance of data quality and governance in training AI models. High-quality data is essential for accurate risk assessment and claims processing. Additionally, ethical considerations, such as fairness and transparency in AI algorithms, must be addressed to prevent biases and discrimination. This aligns with the work of Mullins et al. (2021) and Pisoni and Dìaz-Rodrìguez (2023). For policymakers, our study underscores the need for regulatory compliance in AI applications. The European AI Act EU Act (2023) and other relevant regulations provide a framework for ensuring that AI systems in insurance are transparent, accountable, and compliant with data protection laws. Our findings suggest that insurers must categorize their AI systems based on risk levels and adhere to stricter oversight for high-risk systems. our analysis suggests that insurers should prioritize continuous learning and adaptation in their AI models to address evolving risks and market conditions. This aligns with the principles of dynamic governance and innovation highlighted in recent literature (Grossberg, 2020).

### 6.3 Limitations and future research agenda

Despite the comprehensive nature of our study, there are several limitations that future research should address. Firstly, our review focuses on specific insurance domains (automotive, health, and property), leaving other domains such as life insurance and cyber insurance underexplored. Future research should extend the analysis to these areas to provide a more holistic view of AI applications in insurance.

Secondly, while we discuss the theoretical implications of our findings, further empirical studies are needed to validate the practical impact of AI on risk governance and sustainability in the insurance sector. This aligns with the call for more empirical evidence in the work of Habib and Mourad (2024).

Thirdly, our study identifies significant research gaps in areas such as policyholder behavior analysis in property insurance and claims automation in health and property sectors. Future research should focus on these gaps to provide actionable insights for insurers and policymakers.

Lastly, the rapid evolution of AI technologies necessitates continuous updates to our understanding of their applications in insurance. Future research should explore emerging technologies such as large language models (LLMs) and their potential impact on insurance processes, as highlighted by Balona (2023) and Qiu et al. (2023).

## 7 Conclusions

This systematic review provides a comprehensive overview of the current state of AI in the insurance industry, focusing on automotive, health, and property insurance domains. This paper analyzes 65 academic papers and 33 industry sources. It addresses research questions concerning recent research contributions, industry adoption trends, and key challenges in integrating AI into insurance processes. To do that this paper follows a PRISMA methodology for systematic review. This analysis provides valuable insights into the current state and future directions of AI applications in the insurance industry.

Addressing research gaps and capitalizing on emerging opportunities will be crucial as the insurance industry embraces AI-driven solutions. The paper identifies several critical considerations. These include data quality and governance, ethical implications, regulatory compliance, the need for explainable AI models, and societal acceptance of AI-driven insurance practices. These challenges underscore the importance of responsible AI development and deployment in the insurance sector. The patterns observed highlight the multifaceted nature of technological innovation and the need for sector-specific strategies. This is important for both academic scholars and industry practitioners to drive meaningful advancements in AI-driven insurance. This review bridges the gap between academic research and industry practices. It offers valuable insights for researchers, policymakers, and insurance professionals. As AI continues to transform the insurance landscape, this systematic review can serve as a foundation for future research and informed decision-making.
